# Untargeted metabolomic analysis of tomato pollen development and heat stress response

**DOI:** 10.1007/s00497-017-0301-6

**Published:** 2017-05-16

**Authors:** Marine J. Paupière, Florian Müller, Hanjing Li, Ivo Rieu, Yury M. Tikunov, Richard G. F. Visser, Arnaud G. Bovy

**Affiliations:** 1 Plant Breeding, Wageningen University and Research Centre, PO Box 386, 6700 AJ Wageningen, The Netherlands; 2 Molecular Plant Physiology, Institute for Water and Wetland Research, Radboud University, Heyendaalseweg 135, 6525 AJ Nijmegen, The Netherlands

**Keywords:** Pollen development, Metabolomics, Untargeted analysis, Heat stress, High temperature

## Abstract

***Key message*:**

**Pollen development metabolomics.**

**Abstract:**

Developing pollen is among the plant structures most sensitive to high temperatures, and a decrease in pollen viability is often associated with an alteration of metabolite content. Most of the metabolic studies of pollen have focused on a specific group of compounds, which limits the identification of physiologically important metabolites. To get a better insight into pollen development and the pollen heat stress response, we used a liquid chromatography–mass spectrometry platform to detect secondary metabolites in pollen of tomato (*Solanum lycopersicum L*.) at three developmental stages under control conditions and after a short heat stress at 38 °C. Under control conditions, the young microspores accumulated a large amount of alkaloids and polyamines, whereas the mature pollen strongly accumulated flavonoids. The heat stress treatment led to accumulation of flavonoids in the microspore. The biological role of the detected metabolites is discussed. This study provides the first untargeted metabolomic analysis of developing pollen under a changing environment that can serve as reference for further studies.

**Electronic supplementary material:**

The online version of this article (doi:10.1007/s00497-017-0301-6) contains supplementary material, which is available to authorized users.

## Introduction

Sexual reproduction is a critical process in the plant life cycle and results in the production of seeds and fruits, major components of the human diet. The plant life cycle can be divided into two phases, sporophytic and gametophytic. In angiosperms, the embryo sac is the female gametophyte and is embedded within the ovule, whereas the pollen grain is the male gametophyte and is located inside the anther (Drews and Yadegari 2002; Borg et al. 2009). Pollen development is a complex process that ends with the release of mature pollen grains from the anthers at flower anthesis (Twell 2002; Honys et al. 2006; Hafidh et al. 2016). A major event during pollen development is the meiosis of the pollen mother cell, which results in formation of a tetrad of haploid microspores. Microspores are then released from the tetrads, and during further microspore development, the vacuole expands and the nucleus migrates to one side of the cell. This polarization is the signal for the nucleus to undergo an asymmetrical mitotic division and produce the early bicellular pollen. The two cells of the bicellular pollen have different forms as well as different functions: the smaller, generative cell will later on give rise to the two sperm cells, whereas the surrounding, larger, vegetative cell will produce the pollen tube to ensure delivery of the sperm cells to the female gametophyte.

Tomato (*Solanum lycopersicum* L.) is an economically important crop. Pollen development of this plant is susceptible to various abiotic disturbances (Domínguez et al. 2005; Sato et al. 2000, 2006; Kamel et al. 2010). The development of mature and fertile pollen is one of the key processes for successful fertilization. A decrease in pollen fertility has major consequences for fruit yield (Kartikeya et al. 2012). Pollen development is particularly sensitive to high temperatures (Bokszczanin et al. 2013). A few degrees above the optimal growing temperature of tomato (18–25 °C) can already lead to a decrease in pollen viability. This is often associated with aberrations occurring during pollen development such as premature degeneration of the tapetum and inhibition of anther dehiscence (Suzuki et al. 2001; Matsui and Omasa 2002). In addition, the decrease in pollen viability upon heat stress is associated with a reduction in specific metabolites, such as carbohydrates and polyamines (Pressman et al. 2002; Song et al. 2002).

During its development, the young developing pollen is nurtured with metabolites coming from the tapetum and the locular fluid. Studies with specific mutants, sterile lines and biosynthetic inhibitors have shown that a decrease in the level of particular metabolites, such as the amino acid proline, glutathione, polyamines, and certain hormones is associated with a decrease in pollen fertility. These effects could be (partly) complemented through addition of the respective metabolites (Mattioli et al. 2012; Zechmann et al. 2011; Falasca et al. 2010; Cheng et al. 2006; Goto and Pharis 1999; Ishiguro et al. 2001). Besides their role in pollen nutrition or signalling, metabolites can serve as protectants against environmental stresses. For instance, flavonoids and polyamines can act as scavengers of reactive oxygen species (ROS) (Rice-Evans et al. 1996; Ha et al. 1998). Metabolites such as lipids, flavonoids and polyamines are also involved in the development of the pollen wall, including the different layers of the coat (e.g. exine, intine, sporopollenin) that plays a crucial role in the protection from abiotic stresses (Shi et al. 2015). Despite these examples, the current knowledge on the role and importance of primary and secondary metabolites during pollen development is still limited (reviewed by Paupière et al. 2014).

Most studies addressing metabolic changes during pollen development have focused on the detection of a restricted group of target compounds. Over the last decade, the use of mass spectrometry-based metabolomics approaches, e.g. gas chromatography–mass spectrometry (GC–MS) and liquid chromatography–mass spectrometry (LC–MS), made it possible to detect simultaneously hundreds to thousands of metabolites in a single extract. This has provided a more comprehensive insight in various aspects of plant development and stress responses (Kaplan et al. 2004; Kim et al. 2007; Osorio et al. 2011), including the dynamics of the primary metabolome during pollen germination in lily (*Lilium longiflorum*) (Obermeyer et al. 2013).

Our current understanding of the physiological processes occurring during tomato pollen development under optimal conditions and in response to heat stress is largely based on proteomics and transcriptomics data (Chaturvedi et al. 2013; Honys and Twell 2004). Metabolomics approaches are necessary to complement the omics-derived knowledge and enable developing models for the system as a whole. The objective of this study was to explore the composition and dynamics of the secondary metabolome of tomato pollen, under a normal temperature of 22 °C and after a short heat stress of 2 h at 38 °C. Our results show that the most significant metabolic changes involved the conjugation and relative abundance of flavonoids, polyamines and alkaloids.

## Materials and methods

### Plant materials and growing conditions

Tomato (*Solanum lycopersicum* L.) seeds, cultivar Micro-Tom, were obtained from the National Bioresource Project in Japan (TOMJPF00001). Plants were grown in a climate chamber (MC1600 Snijders Labs, The Netherlands) under constant temperature of 22 °C, with 12-h:12-h photoperiod and a relative humidity of 60%. Light was provided by LED lamps (Philips Green Power LED DR/B/FR 120, ≈250 μmol/m^2^s).

When approximatively five to eight flowers had appeared on the plants, they were subjected to a heat stress of 38 °C or kept at control conditions. After 2 h of treatment, pollen was harvested as described below. Treatments were performed in a staggered fashion, with 30-min gaps between plants to reduce the time that samples were kept on ice during pollen isolation and were done over a 6-day period. Each day was either a control or a heat stress condition; hence, a day was considered as a biological replicate of a condition. A biological replicate consisted of a pool of pollen derived from flower buds of ten plants; hence, each plant was treated only once and three biological replicates were collected for each of the heat stress and the control condition.

### Determination of pollen developmental stages

**Fig. 1:**
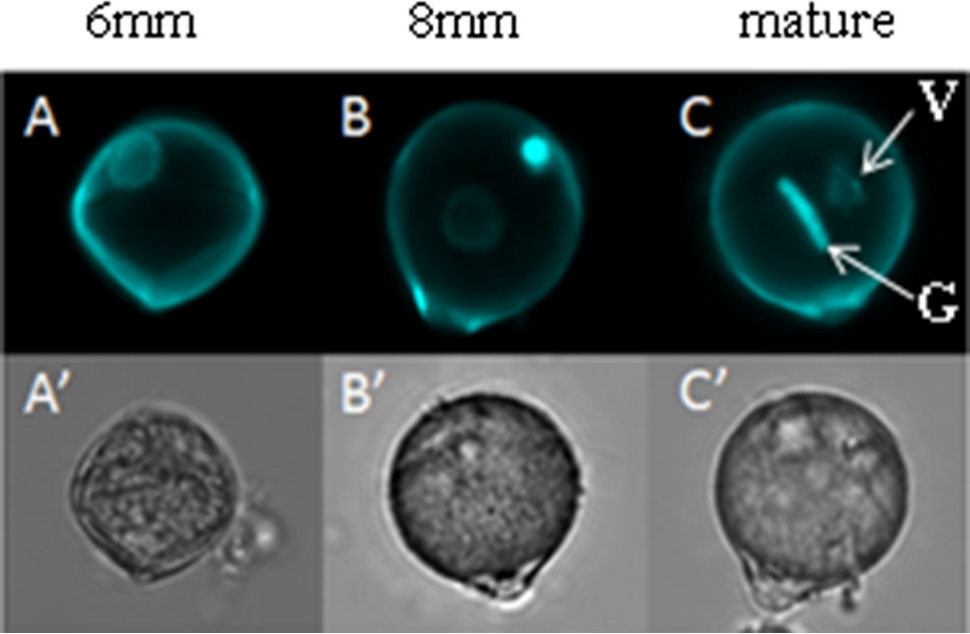
Pollen development of *S. lycopersicum* Micro-Tom cv. Pollen of polarized microspore stage is represented in (**A**)–(**A**′); pollen of early bicellular stage is represented in (**B**)–(**B**′); pollen of mature pollen stage with a vegetative nucleus (*V*) and a generative nucleus (*G*) is represented in (**C**)–(**C**′). Sizes of the anther are indicated above the pictures. **A**–**C** fluorescence microscopy after DAPI staining to visualize the nucleus; **A**′–**C**′ light microscopy

To determine pollen developmental stages, flower buds of Micro-Tom were measured, and anthers were cut into 2–3 pieces and subsequently placed in a 0.3 M mannitol solution. Pollen was released from the anther by vortexing, precipitated by centrifugation at 1000 rpm and incubated with 70% ethanol at room temperature for 30 min. The supernatant was removed after 1 min of centrifugation, and pollen was incubated with 10–30 μl of DAPI 2–5 μg/ml in the dark for 1 h. One droplet of DAPI-stained pollen suspension was transferred to a glass slide and analysed with a Leica TCS SP2 AOBS Confocal Laser Scanning Microscope. Three pollen developmental stages were used for this study: polarized microspore, early bicellular pollen and mature pollen (Fig. 1).

### Pollen harvesting

The pollen harvesting procedure was adapted from the protocol of Firon et al. (2006). Flower buds were removed from the plants and kept on ice. The bud size was determined with a ruler from the base of the anther until the tip including the sepals. Petals, pistil and sepals were removed with forceps before the anther cone was cut into pieces. Anther pieces were transferred into a 1.5-ml Eppendorf tube containing 500 μl ice cold germination solution and stored on ice. The germination solution consisted of 1 mM KNO_3_, 3 mM Ca(NO_3_)_2_.4H_2_O, 0.8 mM MgSO_4_.7H_2_O and 1.6 mM H_3_BO_3_ dissolved in distilled water. Anthers were squeezed with a 1-ml pipette tip to release the pollen. After vortexing, the solution was filtered through four layers of miracloth (Calbiochem) and then centrifuged at 300 g for 2 min at 4 °C followed by a short spin at maximum speed of 17,000 g. Supernatant was removed, and the pollen pellet was washed with 100 μl of ice cold germination solution, followed by centrifugation. This was repeated once, and then the pollen pellet was transferred into a pre-weighted 2-ml Eppendorf tube, frozen in liquid nitrogen, stored at −80 °C and then freeze dried. Pre-weighted 2-ml Eppendorf tubes containing the freeze-dried pollen were subsequently weighted to determine the weight of the pollen.

### Pollen viability

Pollen quality was analysed by in vitro pollen germination and pollen viability tests. Flower buds of 6 and 8 mm were treated as described above, labelled and analysed upon anthesis. Open flowers were analysed directly after treatment. Five plants were used per treatment and stage; per plant one to four open flowers were analysed. Petals, pistil and sepals were removed, and the anther cone was cut into slices and incubated in a humid atmosphere at room temperature for 30 min to allow slow hydration of dry pollen. Anther pieces were transferred to an Eppendorf tube containing germination solution, as described above, supplemented with 5% sucrose and 25% polyethylene glycol 4000. The sample was vortexed for 10 s to release pollen and incubated for 2 h at room temperature while rotating slowly. Per open flower at least 100 pollen grains were counted and scored as germinated, viable or dead. Pollen was considered as germinated when the pollen tube length exceeded the pollen diameter, as viable when pollen was hydrated and as dead when pollen grains did not hydrate. A plant was taken as a biological replicate, leading to at least five biological replicates per developmental stage and per treatment.

### Metabolite extraction

Semi-polar metabolites extractions were carried out at room temperature using water/methanol/chloroform separation, as previously described by Wahyuni et al. (2013). 300 μl of 70% methanol was added to each pollen extract as well as three 2-mm stainless steel beads. Samples were homogenized for 15 min using a TissueLyser (Qiagen^®^) followed by sonication for 10 min and centrifugation for 10 min at maximum speed of 17000 g. 200 μl of the supernatant was transferred into a new 1.5-ml Eppendorf tube containing 200 μl of 70% methanol and filtered with a 0.2-μm polytetrafluoroethylene filter. The weight of original freeze-dried pollen samples varied from 1.96 to 4.8 mg. To avoid a situation where the sample weight-related quantitative metabolic differences would go beyond the linear detection range of the mass spectrometer detector, each extract was diluted with 70% methanol proportionally to the difference between the weight of its original freeze-dried sample and the sample with the minimal weight. The final extract was transferred into a 2-ml crimp glass vial with insert.

### Metabolic profiling

Semi-polar metabolites were separated using a C-18 reversed phase liquid chromatography column and detected by quadrupole time of flight mass spectrometry (LC–QTOF–MS) with negative electrospray ionization. The LC–MS was also coupled to a photodiode array detector allowing spectrophotometric detection. 10 μl of extract was injected and separated using a binary gradient of water (A) and acetonitrile (B), both acidified with 0.1% formic acid, with a flow rate of 0.19 ml/min. The initial solvent composition consisted of 95% of A and 5% of B, increased linearly to 35% A and 65% B in 45 min and maintained for 2 min. The column was washed with 25% A and 75% B for 5 min and equilibrated to 95% A and 5% B for 2 min before the next injection as previously described by De Vos et al. (2007) and Wahyuni et al. (2013). The data were recorded with MassLynx software.

### Metabolite data processing

LC–QTOF–MS data were processed using MetAlign software (available from www.metalign.nl) to correct for the baseline and noise and to perform a mass spectral alignment of chromatograms as previously described by Tikunov et al. (2005) and De Vos et al. (2007). MetAlign output was reduced by omitting mass data showing values lower than the detection threshold (20 ion counts) in more than two samples once the estimated peak signal was subtracted to the estimated noise signal. Compound mass spectra and quantitative ions were extracted from the modified MetAlign outputs using a method described in Tikunov et al. (2012) by MSClust software (available from www.metalign.nl). MSClust output was reduced by keeping compounds that were quantitatively present in all the replicates of one of the experimental treatments: heat stress or control. LC–QTOF–MS masses were kept for analysis when at least one sample had a relative abundance higher than 200 counts. If a quantitative ion automatically selected by MSClust showed saturation of the MS detector, this ion was replaced by its second or third isotopic ion. MSClust output files were then used for compound annotation. Putative annotation of ions was performed with an in-house metabolite database and metabolite online databases Dictionary of Natural Products (http://dnp.chemnetbase.com/) and METLIN (http://metlin.scripps.edu/). The annotation of compounds was performed according to the Metabolomics Standards Initiative requirements (Sumner et al. 2007). Identified compounds were annotated level I when NMR was performed on annotated compounds from the in-house library, level II when an analytical standard was used to annotate the compounds from the in-house library, or when a tandem mass spectrometry was performed and level III when compounds were annotated based on their mass. Annotation level of compounds is indicated in Supplementary data Table 1.

### Tandem mass spectrometry

An MS^3^ analysis was performed using Acquity UPLC–PDA e Detector (Waters) coupled to LTQ Orbitrap XL mass spectrometer (Thermo Scientific). The MS^3^ analysis was performed as previously described by van der Hooft et al. (2011) and Wahyuni et al. (2011). Negative masses 598.25, 612.27, 582.26 and 785.35 have been submitted to MS/MS and MS^3^ using the most intense ion within a 3 Da window around the selected masses, a CID activation type and a normalized collision energy of 35.0. Fragmentation outputs were analysed in Excalibur to establish a fragmentation tree of each mass and allow the identification.

### Determination of flavonoids and polyamines total abundance

The UV spectrum was obtained from the photodiode array detector of the LC–QTOF–MS. Peak areas of flavonoids were obtained by integration at 340 ± 15 nm. Peak areas of conjugated polyamines were obtained by integration at 260 ± 15 nm.

### Statistical analysis

All the statistical analyses were performed with GenStat 18th Edition except mentioned otherwise. For the metabolomics analysis, three biological replicates per developmental stage and treatment were used. Statistical analyses were performed on log2 transformed values. A univariate ANOVA analysis was performed for each annotated metabolite with a treatment structure: condition factor *x* development factor and a block structure considering the number of observations within plants. This block structure was introduced in order to overcome the dependency between stages, since the samples at the three developmental stages were taken from the same pool of plants. Due to the large number of variables generated by metabolomics analysis, 41 secondary metabolites, the statistically significant *p* value threshold was adjusted for multiple testing. A *p* value of 0.01 was used as a threshold. The ANOVA of metabolites with a statistically significant *p* value was followed by a Bonferroni’s post hoc test to correct for the number of analysed pair-wise comparisons; *p* values lower than 0.05 were considered statistically significant. A principal component analysis was performed on log2 transformed and mean centred values for both metabolomics platforms with GeneMaths XT v.2.12. For the short heat stress pollen viability test, at least five biological replicates per developmental stage and per treatment were used. The IBM SPSS statistic software package 20 (www.ibm.com) was used to perform the ANOVAs on the ratio of viable and germinated pollen followed by a Tukey’s post hoc test; *p* values lower than 0.05 were considered statistically significant.

## Results

### Pollen viability

**Fig. 2:**
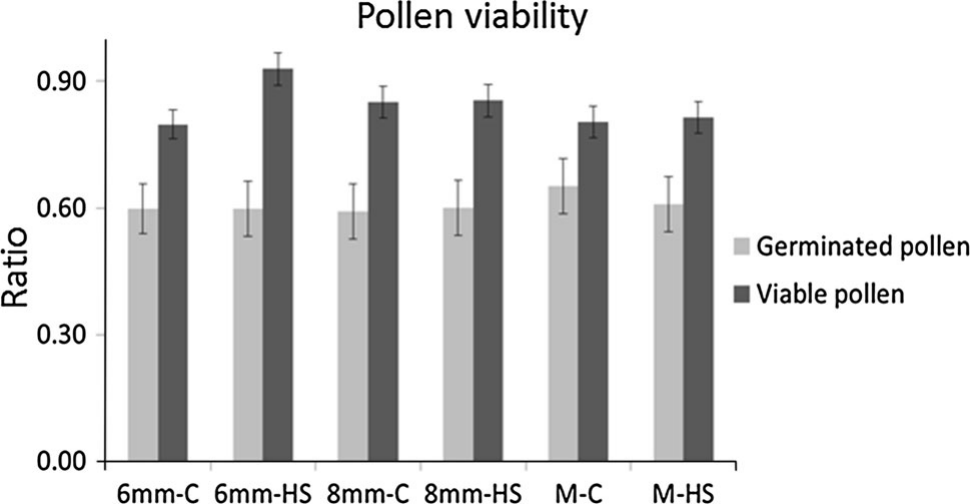
Pollen viability under control and heat stress treatments. 6 mm, polarized microspore; 8 mm, early bicellular pollen; *M* mature pollen, *C* control condition, *HS* heat stress treatment. No statistically significant differences were found between control and heat stress treatments for each of the developmental stages (Tukey test, *p* value <0.05). *Bars* represent the standard error of the mean

In order to determine the impact of a short heat stress on tomato pollen quality, plants were kept in control temperature or exposed to 38 °C for 2 h. Buds of different developmental stages were labelled and analysed upon anthesis: 6 mm buds, i.e. containing mostly polarized microspores, 8 mm buds, containing early bicellular pollen and open flowers, containing mature pollen (Fig. 1). The heat treatment did not lead to statistically significant changes in the proportion of germinated or viable pollen in the three pollen developmental stages tested (Fig. 2, *p* value >0.05). This means that potential differences in metabolite abundance are likely to reflect the cellular heat stress response, rather than deterioration of (part of) the pollen grains.

### Secondary metabolites

To analyse changes in secondary metabolites during pollen development and upon high-temperature stress, a LC–QTOF–MS analysis was performed on semi-polar extracts of tomato pollen. In total 41 putative compounds were detected in different pollen samples of which 38 could be annotated. Most of the putatively identified secondary metabolites belonged to three major groups: flavonoids, polyamines and alkaloids (Supplementary data Table 1). Polyamines showed a large structural diversity, and their peaks were the most intense in the chromatograms (Supplementary data Figure 1). To shed light on the structural variation of the polyamines in tomato pollen, the major parent ions of 598.25, 612.27, 785.35 and 582.26 Da, representing the most abundant unknown polyamines, were subjected to MS^3^ fragmentation (Table 1). We found that spermidine was conjugated with coumaroyl (coum), caffeoyl (caff) and feruloyl (fer) moieties, while spermine was conjugated with coumaroyl moieties only. The fragmentation of the mass 598 gave three relevant fragments: 436 [sperm+coum+coum–H]^−^, 452 [sperm+coum+caff–H]^−^ and 332 [sperm+caff+CO–H]^−^, which led to the identification of caffeoyl dicoumaroyl spermidine, C_34_H_37_N_3_O_7_. The fragmentation of the mass 612 gave four relevant fragments: 466 [sperm+fer+coum–H]^−^, 462 [sperm+coum+coum+CO–H]^−^, 316 [sperm+coum+coum+CO–H]^−^, 175 [feruloyl-2H]^−^ and 145 [sperm]^−^, which led to the identification of feruloyl dicoumaroyl spermidine, C_35_H_39_N_3_O_7_. The fragmentation of the mass 785 gave three relevant fragments: 639 [spn+coum+coum+coum–H]^−^, 665 [spn+coum+coum+coum+CO–H]^−^ and 545 [spn+coum+coum+2CO–H]^−^, which led to the identification of tetracoumaroyl spermine. The fragmentation of the mass 582 gave three relevant fragments: 462 [sperm+coum+coum+CO–H]^−^, 436 [sperm+coum+coum–H]^−^ and 316[sperm+coum+CO–H]^−^, which led to the identification of tricoumaroyl spermidine.

**Table 1:** Identification of hydroxycinnamic acid conjugated polyamines by MS^2,3^ identification

Compound	[M–H]^−^	MS2	MS3	Name	Formula
Compound 1	598	358, 436 [sperm+coum+coum–H]^−^, 462, 478, 452 [sperm+coum+caff–H]^−^	358 → 315, 358 → 300, 452 → 332 [sperm+caff+CO–H]^−^, 452 → 316, 462 → 342, 478 → 342. 478 → 358	Caffeoyl dicoumaroyl spermidine	C_34_H_37_N_3_O_7_
Compound 2	612	492, 372, 476, 466 [sperm+fer+coum–H]^−^, 462 [sperm+coum+coum+CO–H]^−^	492 → 299, 492 → 161, 492 → 372, 492 → 316 [sperm+coum+CO–H]^−^, 492 → 342, 492 → 175 [feruloyl-2H]^−^, 492 → 145 [sperm]^−^, 372 → 175 [feruloyl-2H]^−^, 476 → 145, 466 → 320, 462 → 145, 462 → 342	Feruloyl dicoumaroyl spermidine	C_35_H_39_N_3_O_7_
Compound 3	785	545, 639 [spn+coum+coum+coum–H]^−^, 665 [spn+coum+coum+coum+CO–H]^−^	545 → 399, 665 → 545 [spn+coum+coum+2CO–H]^−^	Tetracoumaroyl spermine	C_46_H_50_N_4_O_8_
Compound 4	582	462 [sperm+coum+coum+CO–H]^−^, 436 [sperm+coum+coum–H]^−^, 342	462 → 342, 462 → 316 [sperm+coum+CO–H]^−^, 462 → 299, 436 → 316[sperm+coum+CO–H]^−^, 436 → 273, 342 → 299,342 → 256	Tricoumaroyl spermidine	C_34_H_37_N_3_O_6_

*Sperm* spermidine, *coum* coumaroyl, *caff* caffeoyl, *fer* feruloyl, *spn* spermine

#### Developmental changes in secondary metabolism of Micro-Tom pollen

**Fig. 3:**
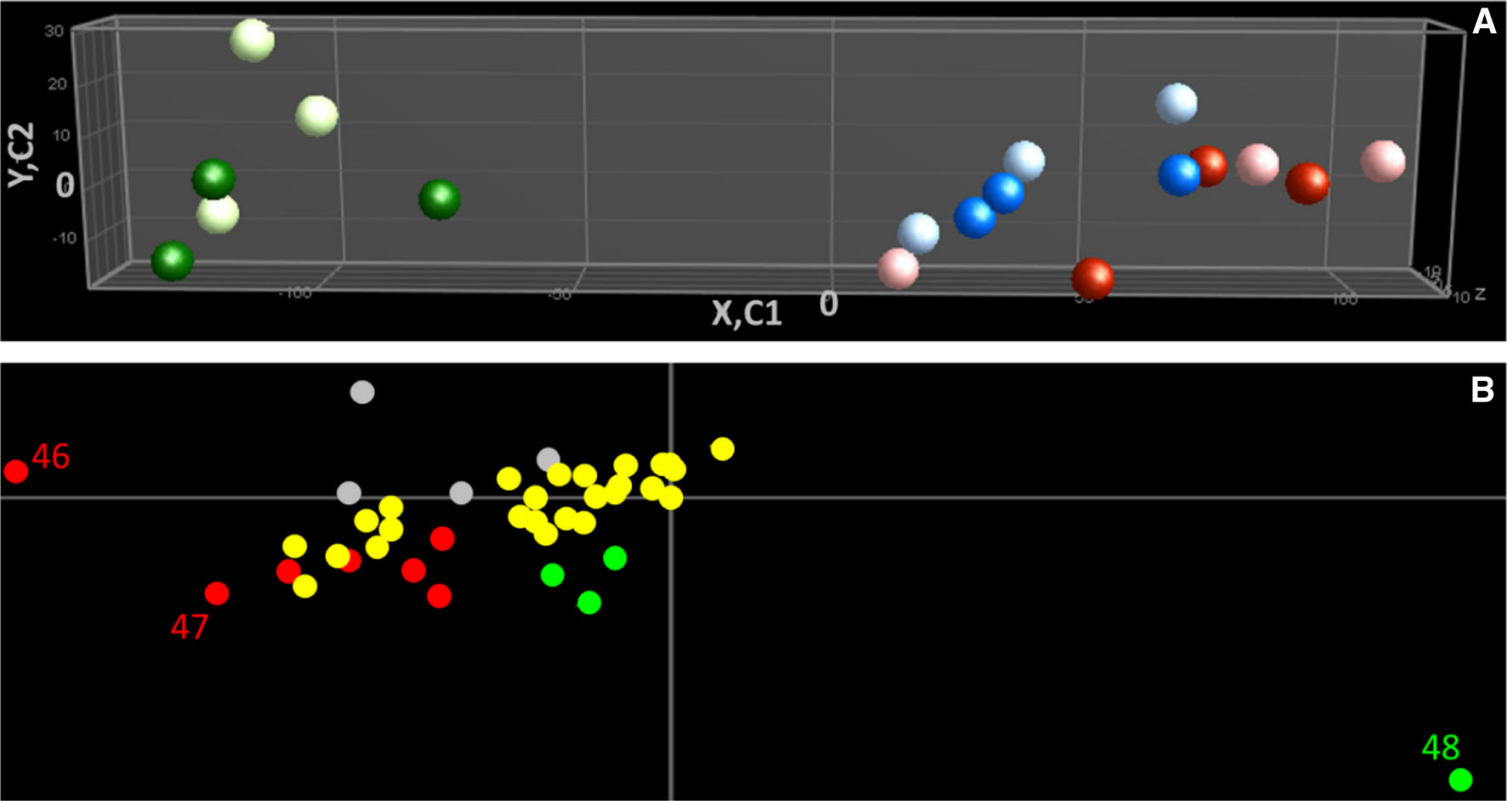
Principal component analysis (PCA) of secondary metabolism. The PCA of the samples is represented in (**a**) with *light green dots* for 6 mm control (C) sample, *dark green dots* for 6 mm heat stress (HS) sample, *light blue dots* for 8 mm-C, *dark blue dots* for 8 mm-HS, *light pink dots* for M–C, and *red dots* for M–HS. Component 1 (C1), component 2 (C2) and component 3 (C3) explain 74.8, 10.3 and 4.3% of the observed variation, respectively. The metabolites responsible for the variation among the samples are represented in (**b**) with *grey dots* for unknown compounds, *red dots* for alkaloids, *yellow dots* for conjugated polyamines and *green dots* for flavonoids. 46, beta-tomatine; 47, tomatine and 48 kaempferol dihexoside. The PCA was performed on log2 transformed and mean centred values

Principal component analysis (PCA) of the LC–QTOF–MS data revealed three groups corresponding to the three pollen developmental stages (Fig. 3a). The first principal component represented most of the differences among the samples explaining 74.8% of the variance. This was due to the difference between the earliest of the three stages, the polarized microspores and the two later developmental stages. The variance of the first component was mainly due to two compounds, the flavonol kaempferol dihexoside and the alkaloid beta-tomatine, which showed a contrasting accumulation pattern during pollen development.

**Fig. 4:**
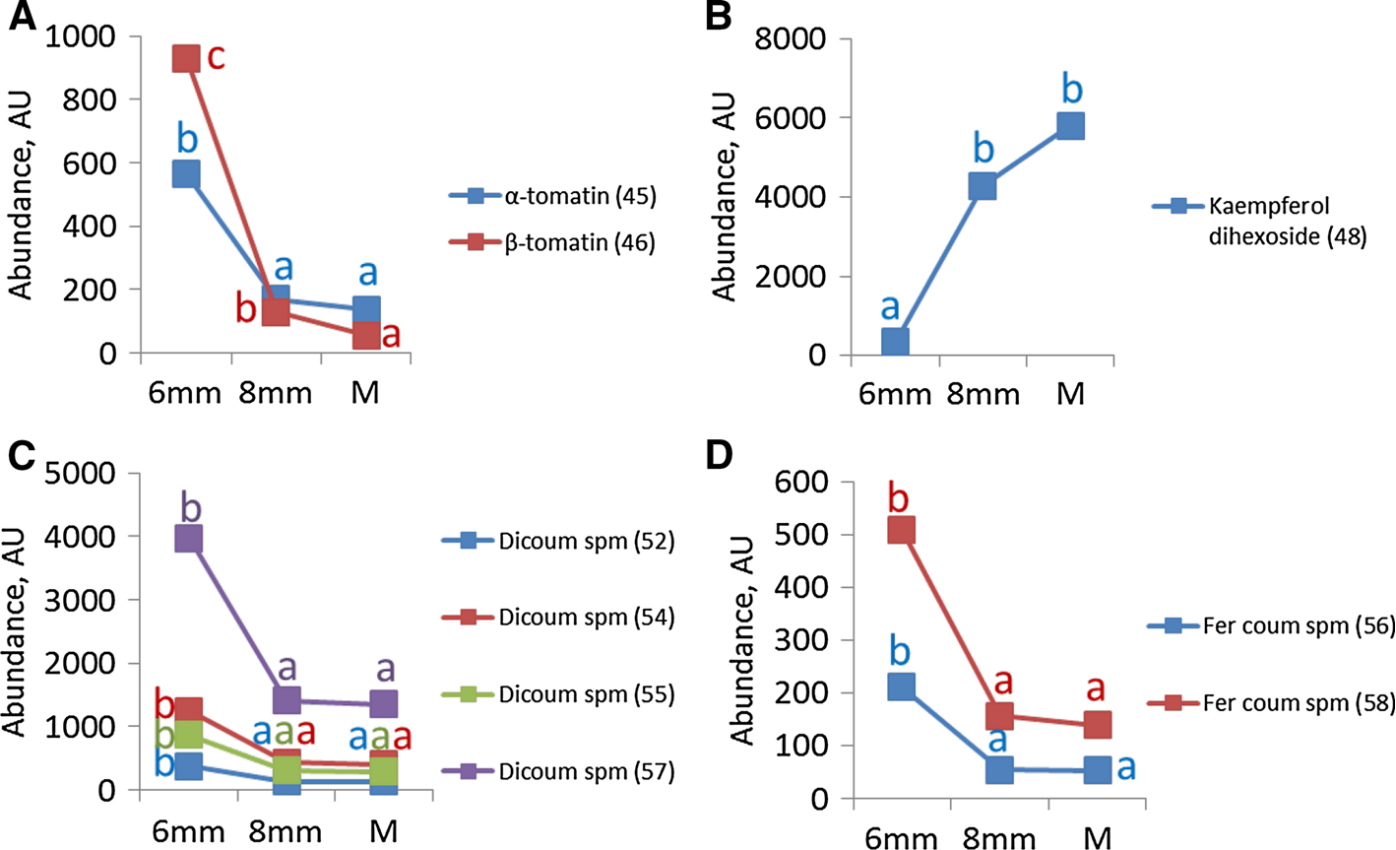
Secondary metabolite profiles during pollen development under control condition. The values per stage represent the average value per stage of both control and heat conditions. Only metabolites showing statistically significant differences between the developmental stages are represented. Abundances of alkaloids are represented in (**a**), flavonoids in (**b**), dicoumaroyl spermidine isomers in (**c**) and caffeoyl dicoumaroyl spermidine isomers in (**d**). 6 mm, polarized microspore; 8 mm, early bicellular pollen; *M* mature pollen, *C* control condition, *Dicoum* dicoumaroyl, *Sperm* spermidine, *Fer* feruloyl. *Letters* show statistically significant differences between the developmental stages per metabolite. *Similar letters* per metabolite indicate that there was no significant difference between the stages. Differences were considered statistically significant when the *p* value of the ANOVA test was lower than 0.01, and the *p* value of the Bonferroni post hoc test was lower than 0.05

Sixteen annotated metabolites showed significant differences between the developmental stages under control conditions (*p* value <0.005, Supplementary data Table 1). During pollen development the alkaloids alpha and beta-tomatine significantly decreased between polarized microspore and mature pollen stage by 2.3-fold and 16.6-fold, respectively (Fig. 4a). Kaempferol dihexoside significantly increased by 16.7-fold in mature pollen compared to polarized microspores (Fig. 4b). Nineteen (out of 25) different spermidine conjugates significantly decreased during pollen development: four isomers of dicoumaroyl spermidine and two isomers of feruloyl coumaroyl spermidine significantly decreased between polarized and bicellular stage (Fig. 4c, d). Different isomers of other conjugated polyamines such as caffeoyl dicoumaroyl spermidine, tricoumaroyl spermidine, diferuloyl coumaroyl spermidine and feruloyl dicoumaroyl spermidine also showed a significant decrease during pollen development (Supplementary data Table 1). In general, the abundance of all the alkaloids and most of the polyamines showed a tendency to decrease during pollen development, although the majority of those differences were not significant (Supplementary data Table 1).

**Fig. 5:**
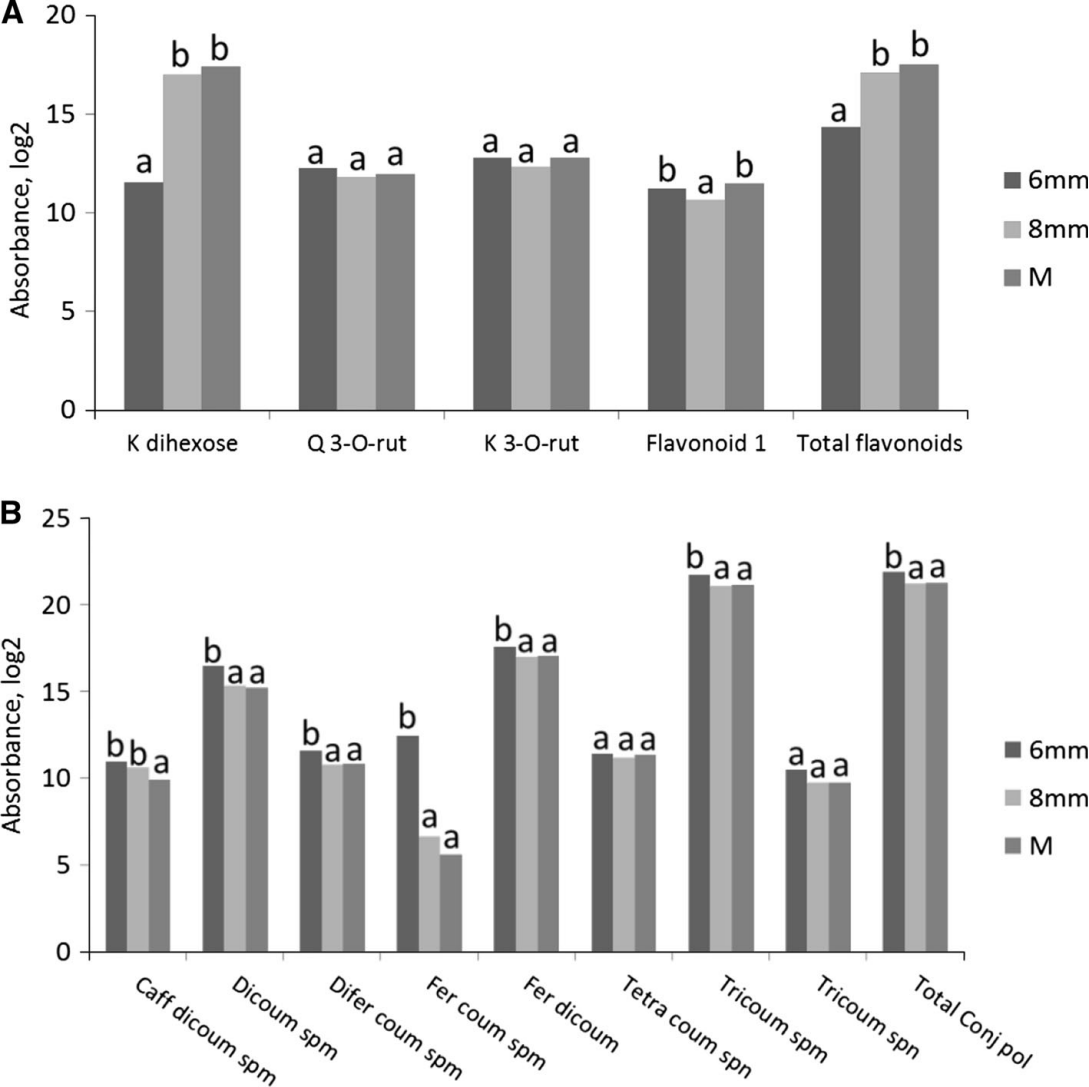
Absorbance profiles of secondary metabolites during pollen development under control condition. The values per stage represent the average value per stage of both control and heat conditions. The absorbance of flavonoids detected using photodiode array (PDA) at 340 ± 15 nm is represented in (**a**). The abundance of polyamines detected by PDA at 260 ± 15 nm is represented in (**b**). Isomers of each conjugated polyamine were summed up to represent the total abundance of each conjugated form. 6 mm, polarized microspore; 8 mm, early bicellular pollen; *M* mature pollen, *C* control condition, *K* kaempferol, *Q* quercetin, *rut* rutinoside, *coum* coumaroyl, *caff* caffeoyl, *fer* feruloyl, *spm* spermidine, *spn* spermine, *total conj. Pol* total conjugated polyamines. *Letters* show statistically significant differences between the developmental stages per metabolite. *Similar letters* per metabolite indicate that there was no significant difference between the stages. Differences were considered statistically significant when the *p* value of the ANOVA test was lower than 0.01, and the *p* value of the Bonferroni post hoc test was lower than 0.05

Differences in ionization efficiency make a quantitative comparison of different flavonoids and spermidines impossible. However, the use of a photodiode array (PDA) detector allowed us to compare the relative abundance of the individual and the total abundance of all flavonoids and polyamines within each sample, by measuring their absorbance at 340 ± 15 nm and 260 ± 15 nm, respectively (Fig. 5, Supplementary data Table 2). During pollen development, the total abundance of flavonoids increased significantly by 8.5-fold from polarized microspore to mature pollen stage (Fig. 5a). Kaempferol dihexoside was the most abundant flavonoid form among the detected flavonoids in mature pollen and significantly increased from polarized microspores to mature pollen. The total abundance of conjugated polyamines decreased with 37% from polarized microspore to early bicellular pollen stage (Fig. 5b). At the level of individual compounds, the total abundance of dicoumaroyl spermidine, diferuloyl coumaroyl spermidine, feruloyl coumaroyl spermidine, feruloyl dicoumaroyl spermidine and tricoumaroyl spermidine forms significantly decreased between polarized and early bicellular pollen stage, while the total abundance of caffeoyl dicoumaroyl spermidine forms significantly decreased from early bicellular to mature pollen stages (Fig. 5b).

#### The effect of a short heat stress on pollen secondary metabolism

**Fig. 6:**
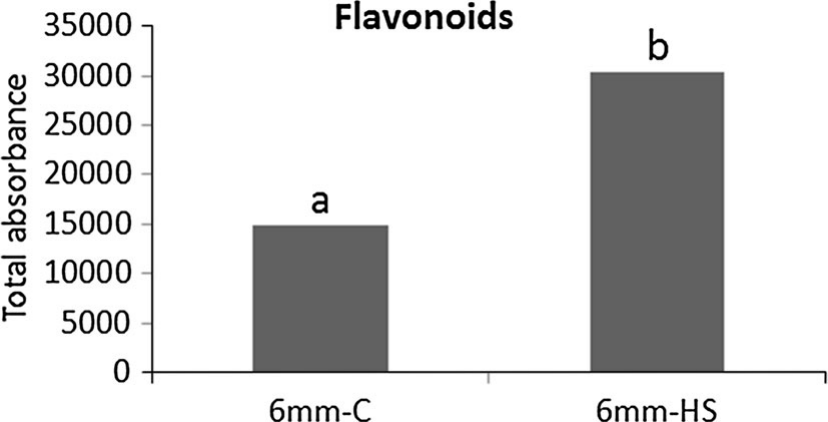
Total absorbance of flavonoids under control and heat stress treatments. The total abundance of flavonoids was determined by the sum of the photodiode array absorbance of individual flavonoids at 340 nm ± 15. *Stars underline* statistically significant differences between 6 mm-C and 6 mm-HS. *Letters* show statistically significant differences between the developmental stages per metabolite. *Similar letters* per metabolite indicate that there was no significant difference between the stages. Differences were considered statistically significant when the *p* value of the ANOVA test was lower than 0.01, and the *p* value of the Bonferroni post hoc test was lower than 0.05

The PCA of the secondary metabolites did not show a clear separation of the two temperature treatments, neither in the first nor in the second or third principle component (Fig. 3a). In line with the PCA, two-way ANOVA revealed that among the 38 putatively annotated compounds, none showed significant differences between control and heat stress (Supplementary data Table 1). However, the total level of flavonoids was significantly, twofold, higher after the short heat stress compared to control conditions in polarized microspores (Fig. 6, Supplementary data Table 3). Although the unidentified flavonoid 1 showed a significant two-way interaction (Supplementary data Table 2), it did not meet the criteria of the Bonferroni post hoc test (Supplementary data Table 3). The individual flavonoids all showed the same trend, but did not reach our statistical threshold. Neither polyamine levels nor alkaloid levels seemed to be affected by the heat stress applied.

## Discussion

The objective of this study was to obtain a broad overview of the changes in the secondary metabolome during tomato pollen development under control conditions and after a heat stress treatment, by using a non-targeted metabolomics approach. Data on secondary metabolites in developing pollen are still limited, and only a few targeted approaches have been used in the past (Paupière et al. 2014).

Metabolomics analyses were performed on three pollen developmental stages: polarized microspores, early bicellular pollen and mature pollen. It is worth mentioning that young, developing pollen, such as at the polarized microspore and early bicellular pollen stages, is tightly enclosed in the anthers and cannot be easily released from them. As was done in previous studies, to achieve the release of early stage pollen, they were collected in an osmotic germination solution (Firon et al. 2006; Pressman et al. 2002; Chaturvedi et al. 2013). However, the incubation of mature dry pollen in a solution during pollen isolation leads to pollen rehydration, and therefore, the studied mature pollen needs to be considered as imbibed pollen. Also, despite the precautions taken during isolation, we cannot exclude that the squeezing of anthers in the germination solution to release young microspores may lead to some contamination from the anther tissue and/or the locular fluid.

### Pollen development

#### Polyamines

Polyamines are known to be widely present in the plant kingdom. They can be found in a free form, bound to proteins or conjugated with other metabolites such as phenolic acids (Aloisi et al. 2016). The majority of the polyamine forms detected in this study of developing pollen were spermidine acylated with hydroxycinnamic moieties such as coumaroyl, caffeoyl and feruloyl groups, commonly known as hydroxycinnamic acid amides. These compounds have also been found in *Arabidopsis thaliana* mature pollen (Handrick et al. 2010). The total level of conjugated polyamines was 37% lower in late pollen developmental stages compared with polarized microspores. This decrease in polyamines correlates with the decrease observed among specific genes and proteins found in the available proteomics datasets of tomato (Chaturvedi et al. 2013) and transcriptomics datasets of Arabidopsis (Honys and Twell 2004) and tobacco (*Nicotiana tabacum*) (Bokvaj et al. 2015), but the whole transcriptome of maturing pollen of tomato from Frank et al. (2009) did not show clear differences between the different developmental stages (personal communication Nurit Firon). In the tomato pollen proteomics data of Chaturvedi et al. (2013), we found two spermidine synthases (Solyc05g005710 and Solyc04g026030) whose abundance decreased during pollen development (Supplemental Table 4). In contrast, however, the abundance of two S-adenosylmethionine synthase proteins (Solyc09g008280 and Solyc10g083970) increased upon pollen development, but these enzymes are not only involved in polyamine biosynthesis, but also play an important role in, for example, ethylene synthesis. In Arabidopsis, expression levels of the gene At1g67990, encoding for a tricaffeoyl spermidine O-methyltransferase involved in the conjugation of polyamines, decreased by 2.5-fold during pollen maturation (Supplementary data Table 4), in line with our own observations at the metabolite level. Unfortunately, we could not find any data for this protein in the tomato proteomics dataset. In tobacco, three early genes of polyamine biosynthesis, two genes encoding for ornithine decarboxylase and one gene encoding for agmatinase had higher expression levels at microspore stage than at mature pollen stage. In summary, both transcriptomics and proteomics data, even from other plant species, support our observation that levels of conjugated polyamines decrease during pollen development and suggest that this is due to decreasing levels of one or more biosynthetic genes and proteins of the polyamine biosynthetic pathway.

The conjugated forms of polyamines are considered to be relatively inactive compared to free forms (Bagni et al. 1994), although the physiological role of acylated polyamines has not been clarified completely yet. It has been suggested, for example, that acylation may promote the stability and compartmentation of polyamines (Bassard et al. 2010), which is of importance for cell types, such as pollen, in which preservation of resources plays a crucial role. Recently, it was shown that a mutation of a spermidine hydroxycinnamoyltransferase in *Arabidopsis thaliana* resulted in a lower level of conjugated spermidine together with pollen wall irregularities (Grienenberger et al. 2009) and defects in seed set (Fellenberg et al. 2008). Our limited understanding of the role of conjugated polyamines in plant development and stress response might, at least in part, be due to the fact that most of the genes involved in their conjugation have not been identified yet (Tiburcio et al. 2014). Nevertheless, several roles of polyamines have been demonstrated. With two to four nitrogen atoms, polyamines may play a role in the nitrogen metabolism, as was already suggested by Altman and Levin (1993). Additionally, polyamines act in the protection against environmental stresses, since they scavenge reactive oxygen species and preserve the integrity of membranes (Ha et al. 1998; Das and Misra 2004). Polyamines are also known to be important compounds for pollen development. For instance, inhibition of the polyamine pathway by pharmacological or genetic means led to a reduction in pollen viability in kiwi (*Actinidia deliciosa*), rice *(Oryza sativa)* and tomato (Falasca et al. 2010; Chen et al. 2014; Song et al. 2001). Finally, polyamines play a role in pollen tube growth through the organization of the cytoskeleton and the cell wall deposition of the pollen tube (Aloisi et al. 2016). A proper cytoskeleton organization is required to ensure the cell expansion and transport of the two sperm cells. Incorporation of polyamines in actin filaments directly affects actin polymerization and subsequent pollen tube growth. In addition, proteins conjugated with polyamines were found in the cell wall of the growing pollen tube, which is another indication that polyamines are involved in pollen tube growth (Di Sandro et al. 2010). It is currently unclear whether the observed changes in the relative abundance of specific polyamine conjugates during pollen development and the small, but significant (37%) decrease in the total level of conjugated polyamines, have functional consequences in relation to pollen development and the subsequent fertilization processes, such as pollen tube growth. It is also relevant to mention that hydroxycinnamic acid amides are not only involved in the conjugation of polyamines but can also be attached to fatty acids to ensure a proper development of the anther cuticle and the pollen sporopollenin (Xu et al. 2017).

#### Flavonoids

We observed a strong increase in the total abundance of flavonoids during tomato pollen development. Kaempferol, conjugated with two yet unknown hexose sugar moieties, was found to be the predominant compound of this class. In line with our study, kaempferol glycosides were the most abundant flavonoid forms in pollen of petunia (*Petunia hybrida L.*) (Zerback et al. 1989). Many studies have shown the importance of flavonoids for pollen viability, especially through the characterization of chalcone synthase (*CHS)* mutants, which show decreased pollen germination in many species, including petunia (Taylor and Jorgensen 1992), maize (*Zea mays*) (Coe et al. 1981) and tomato (Schijlen et al. 2007). The strong accumulation of flavonoids observed in imbibed mature pollen suggests an important role for these compounds in pollen development, pollen germination or pollen tube growth. The quantitative variation of flavonoids we observed could be caused by the activity of genes and enzymes of the flavonoid pathway (Schijlen et al. 2007). In the proteomics data on tomato pollen development (Chaturvedi et al. 2013), only a few proteins corresponding to the flavonoid pathway enzymes were detected, predominantly in the microspore stage only. These were phenylalanine ammonia lyase 6 (PAL6, Solyc10g086180), chalcone synthase 3 (CHS3, Solyc01g090600) and two 4-coumaroyl CoA ligases (4CL, Solyc02g088710 and Solyc12g094520), and their levels were just above the detection threshold (Supplementary Table 4). The low level and low number of flavonoid enzymes detected in this dataset suggest that most flavonoid enzymes accumulate at levels below the detection limit of the proteomics setup used in Chaturvedi et al. (2013) and make it rather difficult to draw conclusions. Unfortunately, there are no transcriptomics data available for developing tomato pollen. The available transcriptomics data of developing Arabidopsis pollen (Honys and Twell 2004) revealed no strong differences between the microspore and bicellular pollen stages in the expression of most of the flavonoid pathway genes, except for one PAL (At3g10340), one hydroxycinnamoyl-CoA shikimate/quinate hydroxycinnamoyl transferase (HCT, At5g48930 (HCT) and one chalcone isomerase (CHI, At5g66220) gene, which were expressed at detectable levels in bicellular pollen, but were not detectable in the earlier microspores. The increasing trend of the expression of these three genes upon pollen development is in line with our metabolic observations in tomato. In tobacco pollen, two differentially expressed chalcone isomerase-like genes were detected, which showed a contrasting expression pattern during pollen development (Supplemental Table 4). It is unclear, however, which of these CHI-like genes encode a genuine chalcone isomerase.

Although the mechanisms of flavonoid action are still unclear, it has been suggested that flavonoids contribute to pollen wall plasticity to allow for fast pollen tube growth (Derksen et al. 1999). Besides, the *Arabidopsis thaliana* mutant *aborted microspores* (*AMS)* showed an alteration in pollen wall with a decrease in genes involved in flavonoid pathways and a decrease in total flavonoid content in flower buds which underlined the importance of flavonoids in the development of pollen wall (Xu et al. 2010, 2014). Flavonoids are also powerful antioxidants that protect against environmental stresses by scavenging reactive oxygen species (ROS) (Rice-Evans et al. 1996). For instance, the rice mutant *mads3* exhibited an increase in ROS in anthers which correlated with a decrease of pollen fertility (Hu et al. 2011). The same mutant showed additionally a metabolic alteration in anthers including the metabolism of carbohydrates, amino acids and Krebs cycle (Qu et al. 2014). Hence, an unbalance ROS homoeostasis in anther had serious effect on reproductive tissues which need to be controlled by a proper antioxidant system including flavonoids. It is important to note that only conjugated forms of flavonoids were detected. These conjugated forms are considered as the storage form of flavonoids. In the petunia *CHS* mutant, pollen germination could only be rescued by adding flavonol aglycones to the in vitro germination medium, while flavonol glycosides were not effective (Mo et al. 1992). It has been hypothesized that flavonol glycosides act as a reserve to provide the aglycone form when needed through the action of a glycosidase. In line with this, we assume that the conjugated flavonoids accumulated in mature pollen will be converted into aglycone forms and used during pollen germination and pollen tube growth. Glycosylation seems to be an important process in the development of the pollen grain since the rice mutant *glycosyltransferase1* responsible for the glycosylation of quercetin failed to produce a mature pollen grain (Moon et al. 2013).

#### Alkaloids

The two glycoalkaloids α- and β-tomatine profoundly accumulated in young polarized microspores compared to mature stages. In *Solanaceae* species the group of glycoalkaloid metabolism (GAME) genes, consisting of approximately 20 genes encoding proteins of different families, have been shown to be the primary machinery of alkaloid biosynthesis (Itkin et al. 2013). Unfortunately, we could only find one aminotransferase-like protein, GAME12, and one 2-oxoglutarate-dependent dioxygenase, GAME11, in the tomato pollen proteomics data (Chaturvedi et al. 2013). Both genes showed a decrease in abundance during pollen development, in line with our observations. In tobacco transcriptomics data, three early genes of the alkaloid biosynthesis pathway-3-hydroxy-3-methylglutaryl coenzyme A reductase (HMGR) and two squalene synthases showed increasing expression levels during pollen development (Bokvaj et al. 2015). It is unknown, however, how alkaloids accumulate in developing tobacco pollen.

Glycoalkaloids are well known for their ability to protect against biotic stresses, and they inhibit the growth of fungi and are toxic for insects (Friedman 2002). The knowledge on the role of glycoalkaloids in pollen is scarce, but the different levels of glycoalkaloids observed in different stages of pollen development might be important to ensure an optimal defence against biotic attacks during plant reproduction. This idea would agree with the finding that the concentration of α-tomatine has been reported to be twofold higher in flowers than in leaves of tomato plants (Kozukue et al. 2004).

In addition to the annotated compounds discussed above, three unknown compounds showed statistically significant differences between the different developmental stages. Elucidating their identities might be relevant to increase the knowledge of the metabolic dynamics occurring during pollen development.

### Impact of heat stress

It is well known that a rise in temperature leads to a decrease in pollen viability (Muller and Rieu 2016). In tomato for instance, both a short heat shock and a long-term mildly elevated growth temperature can lead to a significant reduction in pollen numbers and germination potential (Firon et al. 2006; Dane et al. 1991; Fragkostefanakis et al. 2016). To be able to study the effect of heat treatment on pollen metabolome, we applied a heat treatment that did not affect the viability of resulting mature pollen. Indeed, a more severe heat shock would be required to affect pollen development (Iwahori 1965). However, short-term exposure to non-damaging high temperatures is known to lead to acquired thermotolerance, i.e. improved ability of pollen to withstand subsequent damaging temperatures (Firon et al. 2012; data not shown). Thus, it might be expected that the treatment applied here elicits adaptive metabolic responses in pollen. We did not observe a strong impact of our heat treatment on the pollen metabolome after 2 h of heat stress; an increase in the total abundance of flavonoids in polarized microspore stage was the only significant metabolic alteration detected. Flavonoids play an important role in the detoxification of ROS (Rice-Evans et al. 1996). Under temperature stress, ROS often accumulate and play a role in signalling (Driedonks et al. 2015). However, at the same time their accumulation is harmful for the cell, explaining why temperature stress is often associated with accumulation of ROS scavengers (Suzuki and Mittler 2006). We therefore suggest that the accumulation of flavonoids upon heat is involved in the protection against ROS.

The weak metabolic response in the three pollen developmental stages contrasts with the findings of Kaplan et al. (2004), who showed that a short heat stress of 40 °C led to a dynamic response of the primary metabolome in leaves of Arabidopsis. Given that we analysed the pollen directly after heat application, it is likely that the increase in flavonoids is part of the very initial metabolic response to heat stress and that further responses occur at later time points. The use of earlier developmental stages such as meiotic microspore, known to be most sensitive to short heat stress, could potentially offer a stronger metabolic response. However, the low metabolite content of this stage makes the use of metabolomics analysis challenging (data not shown). As summarized by (Mesihovic et al. 2016) the determination of a heat stress regime is a critical aspect when studying heat stress responses and this strongly influences the final outcome. This study was a first attempt to study the influence of heat stress on the secondary metabolome in pollen. In future studies, we aim to determine the metabolic response of pollen to heat stress at several time points after a given heat treatment and at different developmental stages. This should lead to a more comprehensive picture of the dynamics in the secondary metabolite response of tomato pollen to heat stress.

To summarize, this study was the first attempt to unravel secondary metabolites changes from microspore to mature pollen stage under changing environment and can serve as reference for future investigation of these processes. We performed an untargeted analysis of secondary metabolites in developing tomato pollen grains. Young pollen stages accumulated specific conjugated polyamines and alkaloids, whereas mature pollen stage accumulated more flavonoids. The short heat stress of 2 h at 38 °C led to an increase in total content of flavonoids upon stress in the microspore stage. The accumulation of flavonoids may protect against oxidative damage induced by the temperature increase.

#### **Author contribution statement**

MJP and FM performed the experiments. HL performed the determination of pollen developmental stages. FM did the pollen viability assays. MJP and YMT performed metabolomics analysis. MJP wrote the manuscript. IR, RGFV, YT and AGB co-wrote the manuscript, and AGB headed the project.

## Electronic supplementary material

Below is the link to the electronic supplementary material.

Supplementary material 1 (DOCX 90 kb)
