# Supplementary material for: Untargeted metabolomic analysis of tomato pollen development and heat stress response
Source: Plant Reprod. 2017 May 16;30(2):81–94. doi: 10.1007/s00497-017-0301-6 (PMC5486769; doi:10.1007/s00497-017-0301-6)
Supplement: Supplementary file 1 — Supplementary material 1 (DOCX 90 kb) [file 497_2017_301_MOESM1_ESM.docx]

**Supplementary data Table 1.** Abundance of metabolites detected by the LC-QTOF-MS. Metabolites are organized by classes (alkaloid, flavonoid and polyamine). The average abundance was calculated from the biological replicates per condition (from 6mm-C to M-HS), and the average per stage was calculated (from 6mm to M). Letters show statistically significant differences between the developmental stages per metabolite. Similar letters per metabolite indicate that there was no significant difference between the stages. Differences were considered statistically significant when the p-value of the ANOVA test was lower than 0.01 and the p-value of the Bonferroni post hoc test was lower than 0.05. Boxes represent p-values of the ANOVA test lower than 0.01. N°, compound number; Rt, retention time; An, annotation; ref, reference; 6mm, polarized microspore; 8mm, early bicellular pollen; M, mature; C, control condition; HS, short heat stress condition; Cor, corrected; Stag, developmental stages; Con, condition; Int, interaction stages*conditions. 1,(Iijima et al. 2008); 2,(Moco et al. 2006); 3, (Tikunov et al. 2010); 4,(Roldan et al. 2014); 5, metlin.com; 6, Table 1; 7 (Handrick et al. 2010)

**Supplementary data Table 1 .** *Cont.*

**Supplementary data Table 2** Statistic output of photodiode array data. The average per stage was calculated for each metabolite (from 6mm to M). Letters show statistically significant differences between the developmental stages per metabolite. Similar letters per metabolite indicate that there was no significant difference between the stages. Differences were considered statistically significant when the p-value of the ANOVA test was lower than 0.01 and the p-value of the Bonferroni post hoc test was lower than 0.05. Boxes represent p-values of the ANOVA test lower than 0.01 6mm, polarized microspore; 8mm, early bicellular pollen; M, mature; C, control condition; HS, short heat stress condition; Cor, corrected; Stag, developmental stages; Con, condition; Int, interaction stages*conditions

**Supplementary data table 3**. Fold changes of compounds that showed a statistically significant difference at the two way interaction stage x condition (p-value of the ANOVA < 0.01). Stars indicate statistically significant fold change at the Bonferroni post hoc test. 6mm, polarized microspore; 8mm, early bicellular pollen; M, mature; C, control condition; HS, short heat stress condition

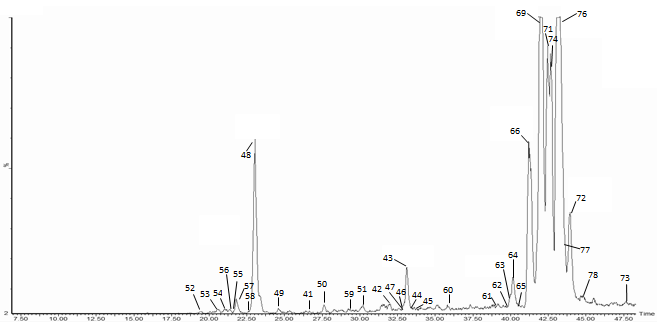


**Supplementary data Figure 1** Total ion count chromatogram of mature pollen obtained by LC-QTOF-MS negative mode. Peaks are labelled with compound numbers that can be found in supplementary data Table 1

**Supplementary data table 4.** Publicly available proteomics data on tomato (Chaturvedi et al. 2013) and transcriptomics data on Arabidopsis (Honys and Twell, 2004) and tobacco (Bokvaj et al. 2015) pollen developement) were mined for genes and proteins involved the polyamine, flavonoid and alkaloid pathways. Two strategies were used to select genes or proteins: (i) gene identities were extracted from the plantCYC and KEGG pathways (polyamines and flavonoids) or extracted from Cardenas et al., 2015. Corresponding orthologs in the three species were found using BLAST analysis (NCBI); (ii) description fields of the omics datasets were searched using keywords for respective metabolic pathway genes. UNM, microspore; BCP, bicellular pollen; S1, microspore; S3, early bicellular pollen; S5 late bicellular pollen,; Mi,microspore; Pol, polarized microspore; M, mature pollen; PAL, phenylalanine amonia lyase; C4H, cinnamate-4-hydroxylase ;HCT, hydroxycinnamoyltransferase; CHI, chalcone isomerase; FLS, flavonol synthase;CHS, chalcone synthase ;4CL, 4-coumaroyl:CoA-ligase;ADC, arginine decarboxylase; AIH, agmatine; NLP, N-carbamolyputrescine amidohydrolase; ARGAH, arginase; SAMDC, adenosylmethionine decarboxylase; SPDS, Spermidine synthase; SHT, 4-coumaroyl CoA N-acyltransferase; TMS, tricaffeoyl spermidine O-methyltransferase; SpeC, Ornithine decarboxylase; SQS, squalene synthase; SAM, S-adenosylmethionine synthase.
